# In pursuit of a valid information assessment method for continuing education: a mixed methods study

**DOI:** 10.1186/1472-6920-13-137

**Published:** 2013-10-07

**Authors:** Soumya Bindiganavile Sridhar, Pierre Pluye, Roland Grad

**Affiliations:** 1 Department of Family Medicine, McGill University, 5858 Côte-des-neiges, 3rd Floor, Suite 300, Montreal, QC H3S 1Z1, Canada

**Keywords:** Content validity, Continuing medical education, Health informatics, Information assessment method, Primary health care, Knowledge translation

## Abstract

**Background:**

The Information Assessment Method (IAM) is a popular tool for continuing education and knowledge translation. After a search for information, the IAM allows the health professional to report what was the search objective, its cognitive impact, as well as any use and patient health benefit associated with the retrieved health information. In continuing education programs, professionals read health information, rate it using the IAM, and earn continuing education credit for this brief individual reflective learning activity. IAM items have been iteratively developed using literature reviews and qualitative studies. Thus, our research question was: what is the content validity of IAM items from the users’ perspective?

**Methods:**

A two-step content validation study was conducted. In Step 1, we followed a mixed methods research design, and assessed the relevance and representativeness of IAM items. In this step, data from a longitudinal quantitative study and a qualitative multiple case study involving 40 family physicians were analyzed. In Step 2, IAM items were analyzed and modified based on a set of guiding principles by a multi-disciplinary expert panel.

**Results:**

The content validity of 16 IAM items was supported, and these items were not changed. Nine other items were modified. Three new items were added, including two that were extensions of an existing item.

**Conclusion:**

A content validated version of the IAM (IAM 2011) is available for the continuing education of health professionals.

## Background

Keeping abreast of medical advances is a challenge, partly because of the turnover of research-based information [1]. In their long-term relationships with patients, family physicians (FPs) strive to combine their clinical expertise with their patients’ preference in arriving at a clinical decision [2]. From time-to-time, clinical decisions are assisted by searches in an Electronic Knowledge Resource (EKR) that provide the FP and their patient with the research-based health information, or evidence, they seek [3]. EKRs can include updated topic summaries, clinical practice guidelines, systematic reviews, or synopses. For example, Essential Evidence Plus contains databases such as POEMs (Patient-Oriented Evidence that Matters) providing access to synopses of new clinical research filtered for relevance to primary care. EKRs can meet information needs such as: addressing clinical questions, supporting decision-making and overcoming the limits of human memory [4]. EKRs enable access to evidence in a timely way, and help to bridge the gap between clinical research and practice, e.g., when they are linked to electronic medical records [5].

There are questionnaires to evaluate user satisfaction with EKRs. However, there is a tool called the Information Assessment Method (IAM), which systematically and comprehensively assesses the value of information from the perspective of the health professional [6]. The IAM is a popular tool for continuing education and knowledge translation in Canada. About 10,000 physicians and pharmacists have participated in IAM-based continuing education programs involving *InfoPOEMS and Highlights*[7-10].

It is employed for documenting the reflective learning of health professionals [11]. The IAM allows health professionals to report the search objective(s), cognitive impact, use and patient health benefit associated with health information retrieved from electronic knowledge resources. These health professionals read health information, rate it using the IAM (brief individual reflective learning activity), and earn continuing education credits. Our research team at McGill University began work on the IAM in 2001 [12]. While IAM items have been used for years, they remain to be validated for content by the users. Therefore, our research question was: what is the content validity of IAM items from the users’ perspective? The purpose of this paper is to present a content validated version of the IAM, which we will call IAM 2011.

The IAM can concurrently assess the following: reasons for an information search, the cognitive impact of retrieved health information, any use of that information, and subsequent expected patient health benefits. When linked to uniquely identified objects of health information, the IAM is a systematic and comprehensive user-centered method to assess the value of information [6]. In its current form, in the context of information retrieval, the IAM questionnaire contains 26 items linked to four constructs: (a) search objective, (b) cognitive impact, (c) the use of information for a specific patient, and (d) information related patient health benefit.

The IAM is based on the ‘Acquisition-Cognition-Application-Outcome’ (ACAO) theoretical model, which we have derived from the work of Saracevic and Kantor in information science [13]. Our ACAO model sequentially integrates the intention to search for health information, related cognitive impacts, corresponding use of this information, and patient health benefit [14].

Acquisition is the process of getting information or objects potentially conveying information, as related to some intentions. Cognition is the process of absorbing, understanding, integrating the information. Application is the process of use (using) this newly understood and cognitively processed information. Outcome is the specific end-result from applying this information.

### Content validation

Content validity is defined as “the degree to which elements of an assessment instrument are relevant to and representative of the targeted construct for a particular assessment purpose” [15]. Content validation of an assessment instrument sometimes involves refinement. The decision to refine elements or items of an instrument or develop new items depends on knowing which are performing poorly. Items might be considered for deletion or modification only if the facets of the targeted construct are not compromised [16].

In measurement, a construct refers to the attributes or variables that are targets of assessment [15]. Thus, each component that the IAM assesses is a construct, e.g., acquisition (search objective). Constructs are composed of facets, which aid in assessing the construct. The term factor is synonymously used with the term facets. We chose not to use the term ‘factor’, because it leads the reader to think of a category of data analytic techniques, such as factor analysis [17]. The items of our IAM questionnaire reflect the facets of each target construct. For each construct, the development of facets was based on literature reviews and empirical studies [6]. We carried out a mixed studies review to (a) identify whether the facets of the four target constructs of the IAM are currently mentioned (either present or absent) in the literature, and (b) identify any additional facets that the IAM does not currently include. The four constructs and their respective facets are presented in Table 1.

**Table 1:** Information assessment method: target constructs and facets

Target construct: Acquisition	Facets: Types of search objectives
	(1) Address a clinical question/problem/decision-making about a specific patient
	(2) Fulfill an educational or research objective
	(3) Search in general or for curiosity
	(4) Look up something I had forgotten
	(5) Share information with a patient/ caregiver
	(6) Exchange information with other health professionals
	(7) Plan, manage, coordinate, delegate or monitor tasks with other health professionals
Target construct: Cognition	Facets: Types of cognitive impacts
	(1) My practice was (will be) changed and improved
	(2) I learned something new
	(3) This information confirmed I did (I am doing) the right thing
	(4) I was reassured
	(5) I recalled something
	(6) I was dissatisfied, as this information had no impact on my practice
	(7) I was dissatisfied as there was a problem with this information
	(8) I disagree with this information
	(9) I think this information is potentially harmful
Target construct: Application	Facets: Types of use of information for a specific patient
	(1) To modify the management of this patient
	(2) To justify or maintain the management of this patient
	(3) To better understand a particular issue related to this patient
	(4) To persuade other health professionals or patients to make changes
Target construct: Outcome	Facets: Types of patient health benefit
	(1) Increasing patient knowledge about heath or healthcare
	(2) Avoiding unnecessary or inappropriate treatment, diagnostic procedure or preventative intervention
	(3) Increasing patient acceptability of treatment, diagnostic procedure or preventative intervention
	(4) Preventing disease or health deterioration (including acute episodes of chronic diseases)
	(5) Improving patient health or functioning or resilience (i.e., how well the patient faces difficulties)

**Figure 1:**
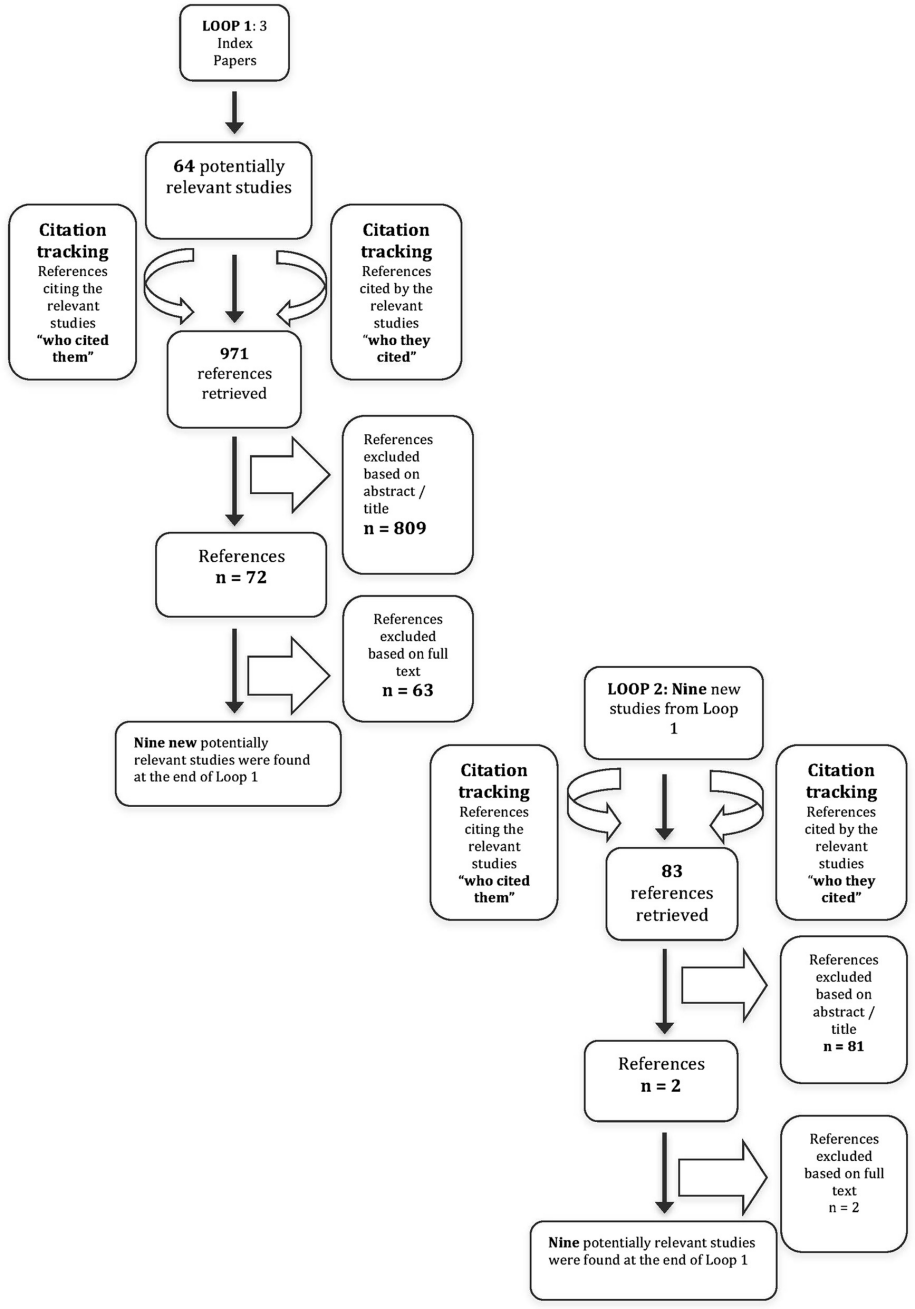
**Flow chart depicting the stages of our literature review.** The nine new references that were found in Loop 1 were used to initiate Loop 2.

Figure 1 depicts our citation tracking search strategy. A deductive-inductive thematic analysis was performed on 73 included studies that used quantitative, qualitative or mixed methods. We coded relevant excerpts from these studies to corresponding themes belonging to IAM facets. The identification of new themes suggested new facets. Thus, all IAM facets tested below were supported by at least one included study.

## Methods

A mixed methods research study was conducted. Mixed methods research is defined as a combination of quantitative and qualitative methods [18]. Indeed, content validation is a mixed methods process that is applicable to all elements of an assessment instrument [15]. Content validity is composed of relevance and representativeness [15]. Relevance is a measure of the appropriateness of the items of an instrument to assess a target construct. Hence, relevant items indicate the essential facets in assessing the target construct. Representativeness refers to the extent to which the elements represent the facet to be assessed. Since relevance is considered a measure, quantitative methods were used to evaluate the relevance of IAM items. Representativeness reflected the extent to which an item clearly represented the facet that was being assessed, hence qualitative methods were used to assess the representativeness of IAM items.

### Design

**Figure 2:**
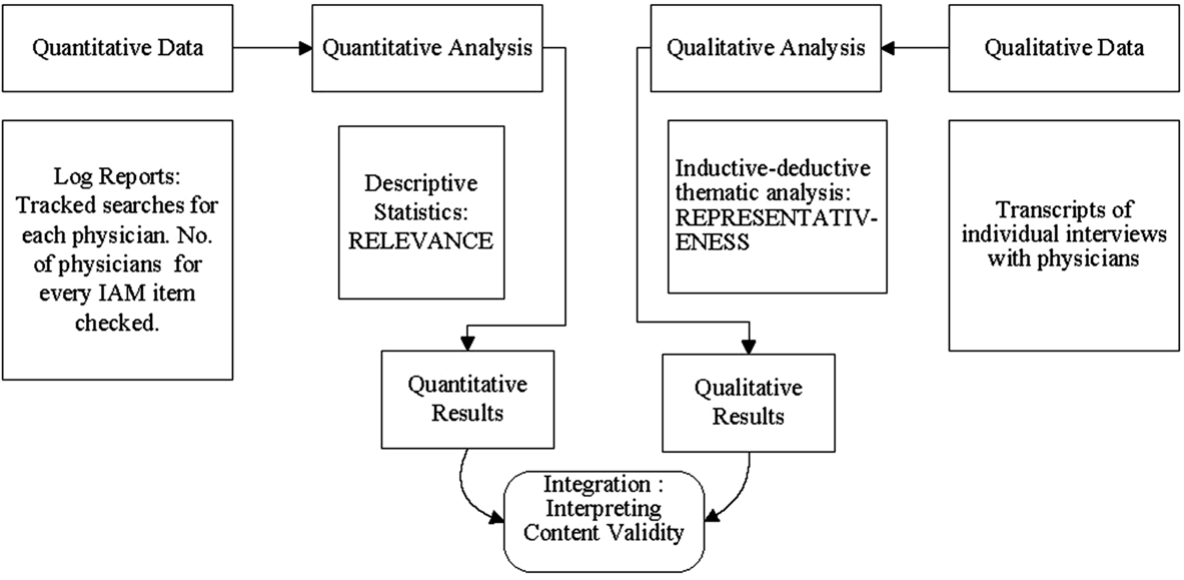
Visual iagram of the mixed methods study design.

Figure 2 represents our mixed methods convergence design. The convergence design obtains different but complimentary data on the same case [19]. The convergence design enables separate data collection and analysis of the same phenomenon, and subsequently the different results are converged during the interpretation stage, by comparing and contrasting quantitative and qualitative results.

### Setting, participants and intervention

Forty Canadian FPs were recruited through personal contacts at various medical meetings or through e-newsletters distributed by the College of Family Physicians of Canada. All participants were offered a handheld computer at no cost, for participating in this study. In exchange for participation, FPs were also offered continuing medical education credits for each search for health information they rated. All participants were practicing FPs. The average age of participants was 44 years; there were 17 women and 23 men. Twenty-eight (68%) had a connection through teaching or research to a faculty of medicine. Participants agreed to rate their searches in Essential Evidence Plus, using the IAM questionnaire. Both Essential Evidence Plus and the IAM software were provided on their handheld device.

### Quantitative data collection/analysis

A longitudinal observational study was conducted, whereby participants searched for information on their handheld computer in routine clinical practice over an average of 320 days. Searches for information contained various information objects, such as synopses of clinical research (POEMs), guidelines and abstracts of Cochrane reviews. When a participant opened one information object and responded to the IAM questionnaire, it was defined as a ‘rated hit’. The IAM software generated log reports which included: (a) date and time of all information searches that included rated ‘hits’, (b) titles of rated hits (the information objects), and (c) IAM item responses by physician linked to specific ‘hits’. The IAM item responses were collected at the search level (search objective) and at the ‘hit’ level (cognitive impact and use). The IAM item responses form the pool of quantitative data. A total of 1,767 rated searches were performed by the participants during the study period which comprised 3,300 rated ‘hits’. Quantitative data analysis for each of the four constructs: Item relevance (*R*) was calculated using the following formula.

$$R=\frac{\mathrm{Number}\;\mathrm{of}\;\mathrm{the}\;\mathrm{items}\mathrm{was}\;\mathrm{rated}\;\mathrm{or}\;\mathrm{explained}}{\mathrm{Total}\;\mathrm{number}\;\mathrm{of}\;\mathrm{rating}\;\mathrm{or}\;\mathrm{explanations}}$$

For items where *R* < 10%, we considered the relevance of that item to be questionable. We chose *R* < 10% as the cut-off for questioning item relevance for two reasons: (a) there is no agreed upon criterion or universal cut off to determine the extent of content validity [20]; and (b) since the IAM items are based on 10 years of research in a focused context, it was likely that many items would be relevant. Hence, in order to identify items that had very low relevance we chose the arbitrary cutoff of 10%. For example, the number of times the item “Address a clinical question” was checked = 1310; the total number of times any of the seven items in the acquisition construct (Search Objectives) was checked = 4253; therefore, R for this item was $\frac{1310}{4253}\times 100=31\%$ and this item was deemed to be relevant.

### Qualitative data collection/analysis

A multiple case study was conducted, where a case was defined as one rated search submitted by one participant. For these rated searches, participants were interviewed to explain a purposeful sample of their rated information ‘hits’. The emphasis was on ‘purposively selecting information-rich’ cases [21]. As a result, the purposeful sample consisted of ‘hits’ where information was used for a specific patient or where information had a negative type of cognitive impact. A participant’s explanation for checking one IAM item, for one information ‘hit’ was defined as a ‘unit’.

The interviewer was an anthropologist experienced in conducting interviews. Interviews were conducted based on a semi-structured questionnaire (See Additional file 1). Individual log reports containing a list of rated searches were e-mailed to each FP as an *aide memoire,* before the interview*.* The interviews were recorded with permission of each participant. Most interviews were conducted by telephone and lasted 30 to 45 minutes. Each participant was interviewed twice during the data collection phase.

During the interview, participants were reminded of their rated search for information using their IAM item responses, in terms of: ‘Search objectives’ and ‘Cognitive impacts’. The participants provided explanations as to why they checked a particular IAM item during a search for information. Additionally, open questions sought explanations as to how the information was used for a specific patient (if applicable) and to describe any patient health benefits (perceived or witnessed during follow-up contact). If the participants described any information use or patient health benefit further questions were asked using a list of items of information use for a specific patient and patient health benefit, following an interview guide.

Qualitative data analysis was used to evaluate representativeness of IAM items. To facilitate this analysis we used specialized software (NVivo7). An inductive-deductive thematic analysis of the interviews was carried out using IAM items as initial themes [22]. Each unit or a physician’s explanation for one rated IAM item was coded to the respective theme based on the IAM item definitions (presented in Additional file 2). There were four possibilities: (1) a unit was a ‘FIT’ when the physician’s explanation was concordant with the definition of the IAM item, (2) ‘MISFIT’ when it was concordant with the definition of another IAM item, (3) ‘UNCLEAR’, when an explanation was provided, but this explanation was not clear, (4)’NEW’ when an explanation did not correspond to any of the current IAM item definitions and was a potential new item, and (5) ‘NONE’, when no explanation was provided. We coded a total of 3,199 units during the thematic analysis process. An item was considered representative if the number of ‘FIT’ units was ≥ 80% of all responses (FIT + MISFIT + UNCLEAR + NEW + NONE). There is no agreed upon criterion for determining the extent to which a measure has attained content validity. Nunnally [23] noted that “inevitably content validity rests mainly on appeals to reason regarding the adequacy with which important content has been cast in the form of test items.”

### Integration of quantitative and qualitative components, and expert panel discussion

The integration of the quantitative and qualitative methods occurred at the stage of interpreting quantitative (relevance) and qualitative (representativeness) results. After this integration, we modified problematic items based on a list of recommendations on how to compose good questionnaire items [24-26]. We subjected a draft version of the proposed IAM 2011 for review by a panel of seven persons comprised of FP researchers, librarians, information scientists and an anthropologist. This was a multidisciplinary panel of researchers with expertise in studying the value of information. Expert panel discussion is a core component of Content Validation Guidelines [15]. Nunnally and Bernstein [27] also noted that results from such an exercise can guide judgments about the content validity of any items that need modification or need to be omitted. The panel members evaluated and suggested changes for each item given its relevance to the construct, representativeness to the item definition, clarity, language, and response format.

Based on opinions from this expert panel, final changes were made, and the IAM 2011 questionnaire was created.

## Results

The relevance and representativeness for each IAM item are presented in Table 2. Sixteen items were both relevant and representative and were retained without modification. For example, item: *To look up something I had forgotten* (Relevance = 16% and Representativeness = 88%). Nine items were relevant but not representative and were considered for modification. For example, item: *I recalled something* (Relevance = 18% and Representativeness = 78%). From our qualitative data analysis we found that participants understood this item as being ‘reminded of something that they already knew’ or had seen before. Hence we suggested a modification of this item. Three items were found to be representative but not relevant. For example, item: *I disagree with this information* (Relevance = 4% and Representativeness = 66%). We suggested removal of the items that were not relevant. We also found one new theme/facet that was suggested by our qualitative data: The *use of information in a discussion with this patient or other health professionals.* This facet of information use was not present in previous versions of the IAM, while it was reported 53 times in 30 searches for information.

**Table 2:** Relevance and representativeness of IAM items

**Items**	**Relevant?**	**Representativ?**	**Decision**
**Reasons for information search**	Number of ratings = 4253		
1. Address a clinical question/problem/decision-making about a specific patient	YES	YES	**Retain**
	31%	98%	
2. Fulfill an educational or research objective	YES	YES	**Retain**
	10%	98%	
3. Search in general or for curiosity	YES	YES	**Retain**
	12%	12%	
4. Look up something I had forgotten	YES	YES	**Retain**
	16%	88%	
5. Share information with a patient/ caregiver	YES	YES	**Retain**
	15%	93%	
6. Exchange information with other health professionals	YES	YES	**Retain**
	12%	97%	
7. Plan, manage, coordinate, delegate or monitor tasks with other health professionals	NO	YES	**Consider removal**
	**5%**	86%	
**Cognitive impact**			
Items of Positive Impact	Number of ratings = 6329		
1. My practice was (will be) changed and improved	YES	YES	**Retain**
	15%	83%	
2. I learned something new	YES	YES	**Retain**
	30%	80%	
3. This information confirmed I did (I am doing) the right thing.	YES	YES	**Retain**
	24%	88%	
4. I was reassured	YES	YES	**Retain**
	23%	90%	
5. I recalled something	YES	NO	**Consider modification**
	18%	78%	
Items of Negative Impact	Number of ratings = 166		
6. I was dissatisfied as this information had no impact on my practice	YES	YES	**Retain**
	47%	83%	
7. I was dissatisfied as there was a problem with this information	YES	YES	**Retain**
	40%	83%	
8. I disagree with this information	NO	NO	**Consider removal**
	**4%**	66%	
9. I think this information is potentially harmful	NO	YES	**Consider removal**
	**8%**	80%	
**Information use for a specific patient**	Number of units = 737		
1. To modify the management of this patient	YES	NO	**Consider modification**
	19%	53%	
2. To justify or maintain the management of this patient	YES	YES	**Retain**
	39%	92%	
3. To better understand a particular issue related to this patient	YES	YES	**Retain**
	28%	97%	
4. To persuade other health professionals or patients to make changes	YES	NO	**Consider modification**
	14%	79%	
**Patient health benefit**	Number of units = 766		
1. Increasing patient knowledge about heath or healthcare	YES	YES	**Retain**
	23%	96%	
2. Avoiding unnecessary or inappropriate treatment, diagnostic procedure or preventative intervention	YES	YES	**Retain**
	21%	88%	
3. Increasing patient acceptability of treatment, diagnostic procedure or preventative intervention	YES	NO	**Consider modification**
	18%	3%	
4. Preventing disease or health deterioration (including acute episodes of chronic diseases)	YES	NO	**Consider modification**
	17%	64%	
5. Improving patient health or functioning or resilience (i.e., how well the patient faces difficulties)	YES	NO	**Consider modification**
	20%	66%	

### Example

Interviewer: “Would you say this information would have any consequences for the patient?”

MD25: “Well, it might, *I would probably have a discussion with the patient* about the therapeutic options if the diagnosis was confirmed, so, you know. (…) they would probably have more information before going ahead to meet a specialist (…).”

The Expert Panel analyzed the results of our mixed methods study and decided to modify eight items; this led to the final version of IAM 2011.

For example, consider the following old item: *There was a problem with this information*. Experts commented that the wording of this item might overlap with ‘dissatisfaction’. Furthermore, the old item does not emphasize problems with respect to structure and amount of information. Thus we modified the item as follows “*There is a problem with the presentation of this information”.*

The panel also suggested that one double barreled item needed to be split into two items. Old item: To justify or maintain the management of this patient. This item was split into two: (a) I hesitated between options for this patient, and I used (will use) this information to justify a choice, and (b) I did not know what to do, and I used this information to manage this patient. Detailed results are available online [28].

## Discussion

**Figure 3:**
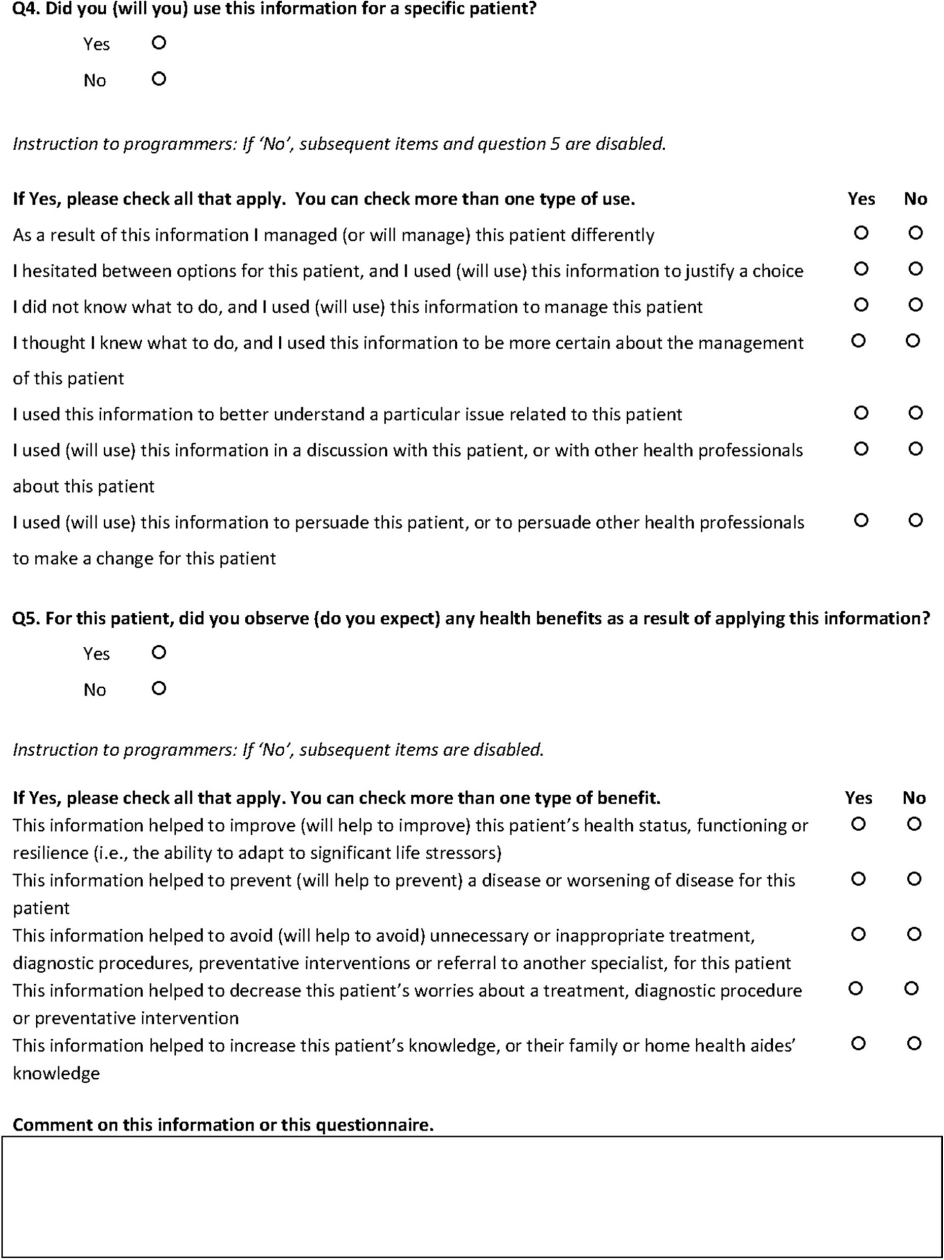
Content validated version of the information assessment method (IAM 2011).

The results from our mixed methods study and Expert Panel Discussion have led us to propose a content validated version of the IAM containing 28 items (seven for Search Objectives, nine for Cognitive Impacts, seven for Information Use for a Specific Patient, and five for Patient Health Benefit) (Figure 3). This mixed methods study helped us identify items that needed modification (Representativeness < 80%) or removal (Relevance < 10%).

Based on the decisions of our expert panel, item modifications were made. However, three items that were considered for possible removal were retained, for the following reasons:

Item: Plan, manage, coordinate, delegate or monitor tasks with other health professionals

In about 1,800 rated searches for information, 4,253 reasons were selected by participants, from which this item was endorsed only 197 times. This suggests its relevance is approximately 5%. Although this item was highly representative, participants did not commonly rate this item as a reason for searching for health information. Participants rated this item if they were teaching or supervising a resident or when they had to collaborate with a nurse, pharmacist, respiratory therapist, subspecialist (e.g., rheumatologist), or a patient’s family doctor. We considered the following two possible causes for the low relevance of this item: (a) the EKR that was used in the study might have contained little information on collaborating with other health professionals around patient care, hence, the frequency of rating this item was low; and (b) the item included more than one reason to search (plan or manage or coordinate or delegate), which is not a best practice. Taking both possible issues into consideration, we retained the item, but with the following modification - “*To manage aspects of patient care with other professionals”*.

*Item: “I disagree with this information”* and *Item:* “*I think this information is potentially harmful”*

These two items of negative cognitive impact had low relevance (4% and 8%). This implies that participating FPs rarely disagreed with the information they retrieved nor did they believe it was harmful. One explanation for this result is that participants used an EKR (Essential Evidence Plus) that contained information filtered for validity and relevance. Thus, harmful or problematic information was rarely identified. Although these two items were not relevant in this study, they can potentially contribute to research on the value of health information and help identify harmful information in other EKRs. Thus we decided to retain these two items of negative cognitive impact.

Our study faced a limitation: the limits of human memory. The number of days between a search for information on the handheld computer and telephone interviews varied widely, from 1 to 250. This issue was addressed by excluding searches that participants could not clearly remember. It is possible that the value of forgotten searches may be different from those that were not forgotten. In addition, participants represented a convenience sample of 40 FPs and their item ratings may be different from those obtained from a larger random sample of FPs. However, a sufficiently large sample of 3,300 rated hits was obtained for the purpose of content validation. Moreover, a representative sample of the targeted population is not needed for content validity assessment. For example, according to Vogt et al. [29], three to six focus groups with five to 10 participants in each group are sufficient.

The strength of our mixed methods content validation study is that the members of the target population (FPs) were consulted as experts when they used the IAM in their routine clinical practice. Participants had the opportunity to use the IAM questionnaire over a long period of time to provide a perspective on IAM items. This longitudinal approach is preferred over a focus group discussion that would have provided only a snapshot of the collective opinion [29].

In future educational research, the IAM can be used to compare EKRs, e.g., Essential Evidence Plus versus DynaMed. The data collected through IAM 2011 can be used for assessing the value of research-based health information from a users’ perspective. Finally, content validity is an integral component of construct validity, and construct validity is the degree to which an assessment instrument measures the targeted construct [15]. Future research should examine the construct validity of IAM 2011 for EKRs, e.g., using factor analysis techniques.

## Conclusion

The content validated IAM 2011 can systematically and comprehensively document reflective learning in medical education, and facilitate the evaluation of education programs. In other words, the IAM is both an intervention at the individual level, and a program evaluation tool at the collective level [30]. At the participant level (individual response), reading information and assessing it with IAM qualifies as a ‘brief individual reflective learning’ activity, which allows the provision of continuing education credits. The IAM is currently being used in various Canadian continuing medical education programs, e.g., in one program, physicians are asked to read and rate their searches for information in databases such as DynaMed. At the program level (all responses from all participants), IAM documents three educational outcomes (participation, learning, performance), while many current program evaluations provide only documentation of attendance.

### Ethical approval

Ethics approval was obtained from the McGill University Institutional Review Board.

## Abbreviations

ACAO: Acquisition-cognition-application-outcome; EKR: Electronic knowledge resource; FP: Family physician; IAM: Information assessment method; POEM: Patient-oriented evidence that matters.

## Competing interests

The authors declare no competing interests.

## Authors’ contributions

SBS carried out the entire study. PP and RG supervised her work and participated in all stages of the research. All authors participated in drafting the manuscript. All authors read and approved the final version of the manuscript.

## Authors’ information

Dr. Soumya Bindiganavile Sridhar, MBBS, MSc, is a Family Medicine Resident at McGill University. Her research interests include questionnaire validation and mixed methods research.

Pierre Pluye, MD, PhD, is FRQS Scientist and Associate Professor, Department of Family Medicine, McGill University. He has expertise in mixed methods research studies and mixed studies reviews. He studies the application of information derived from electronic knowledge resources, and has co-developed the Information Assessment Method (http://www.mcgill.ca/iam/).

Roland Grad, MDCM, MSc, CCFP FCFP, is a Family Physician and Associate Professor, Department of Family Medicine, McGill University. He co-developed the Information Assessment Method, a tool for knowledge translation in primary health care.

## Pre-publication history

The pre-publication history for this paper can be accessed here:

http://www.biomedcentral.com/1472-6920/13/137/prepub

## Supplementary Material

Additional file 1: Interview guide. Click here for file

Additional file 2: Definitions of IAM items. Click here for file
