# Supplementary material for: In pursuit of a valid information assessment method for continuing education: a mixed methods study
Source: BMC Med Educ. 2013 Oct 7;13:137. doi: 10.1186/1472-6920-13-137 (PMC3842783; doi:10.1186/1472-6920-13-137)
Supplement: Additional file 1: — Interview guide. [file 1472-6920-13-137-S1.pdf]

Additional file 1
INTERVIEW GUIDE

PART A. Introduction
(Interviewer presents herself)
So before we begin, maybe I can briefly explain the context of the interview?
As you might already know we are doing this study to document the impact of databases like *Essential Evidence+® (EE+),* and to validate our information impact assessment method. So my plan for today is to review your most recent searches for information and your ratings. When you searched *EE+* and answered questionnaires, a report of your answers was provided to me. So we'll use this report to stimulate your memory. It may not be easy but we'll try to recall the context of a few searches, as well as the relevance, impact and use of the information you found. If you can't remember, it's ok, we will just move on to the next search.

So this interview may last about 60 minutes. Is that ok? (*Reschedule the interview if needed*)
- Before we start, do you have any questions?

GENERAL QUESTIONS
-QA1. Do residents or colleagues use your PDA?
-QA2. Do you use the latest version of IAM & *EE+*?
Probe: If not, can I ask you why?
-QA3. On the report, I see that when you searched with *EE+* on your PDA, X (*Nb of deleted hits*) opened *Essential Evidence+* pages were deleted, so not rated. Can you explain to me why you delete items, in general?
-QA4. On the report, I see that (like most MDs in this study), you only rated a rule calculator (CDSS) X (*Nb of times, e.g., once*) (*Read titles if necessary*). Do you use these on another computer besides your PDA?
-QA5. Do you ever retrieve POEMs using *EE+* on another computer besides your PDA?
Probe: If yes, do you remember retrieving a POEM that you previously received on email?
-QA6. And in general, would you say you prefer pulling information or the information being pushed to you?
QA8 Do you think the push POEMS (on email) had any effect on your pull behavior (*in EE+ or elsewhere*)?
QA7. Do you have any comment to make on the questionnaire?
Probe: Did the length of the questionnaire discourage you to rate information items?

PART B. ACQUISITION (RELEVANCE) Questions at the SEARCH LEVEL
First, I would like to ask you a few questions about a search, that is to say a set of opened *EE+* pages that you retrieved and rated.
-QB1. Do you remember that on (*read date and time*) you did a search on (*read keyword-s*)?

[If needed: you retrieved *(read information hits titles)*?]

- **If NO**: ask if interviewee has residents using the PDA or ask about another search.

-QB2. Did you do this search by yourself or in the presence of someone else?
-QB3. Do you remember where you were when you did this search?
-QB4. (*If clinical situation*) Did you search before, after, or during an encounter with a patient?
-QB5. Can you tell me the story around this search, e.g., do you remember what triggered this search?
Note: *Continue the interview when SQ2 to SQ5 are clearly answered (clear search). Stop the interview about this search when one or more than one of these questions remain(s) unanswered, or when the interviewee maintains that he or she does not remember this search (forgotten search).*
-QB6. According to my report, you searched for the following reason(s) (*read log-report*): (…), and you said that this search (*did* or *did not*) meet your objective(s) (*according to* r*eport*). Is that correct?
-QB7. Can you explain to me what led you to rate (*read each search objective one at a time and wait for answer*)?
-QB8. Did you search in another source of information? For example did you seek information from colleagues, Internet, journals, textbooks, personal notes or library services?

- **If NO**, go to PART B2

-QB9. What was this source (or what were these sources), and what did you find?
-QB10. Was this information (from source X) in agreement with or in conflict with *EE+*?
-QB11. Was this information more relevant, equally relevant, or less relevant compared to *EE+,* given your objective(s)?

PART C. COGNITION = IMPACT = Questions at the HIT LEVEL
Second, I would like to ask you a few questions about the opened *EE+* page(s) that you retrieved and rated in this search. When you did a search on (*read keyword-s*), you opened **X** (*read Nb of hits*) *EE+* pages (*read information hits titles and types, e.g. POEM*).

<u>-QC1. For the **first** *EE+* page entitled (*read title*), you reported the following impacts (*read log-report*):</u> (…). In what specific ways did this page have the following impact "(*read impact*)"?

- *Repeat the question for each impact*

(*According to log report, ask QD1 or QD2-QD3-QE1-QE2*)

PART D. APPLICATION = USE = Questions at the HIT LEVEL (or search level, if all hits used the same way)

NO	-QD1. You reported that this EE+ page was NOT applied for a specific patient.<br>But even though you didn't use it for a specific patient, did you use it in any other way?

<table>
<tr><td>U
S
E</td><td>PROBE. For example, would you say that this EE+ page changed your (awareness) or (thinking) or (understanding) of a specific issue?
Then ask HQ1 second hit</td></tr>
</table>

OR

<table>
<tr><td rowspan="2">U
S
E</td><td>-QD2. You reported that this EE+ page was applied for a specific patient.
Was this information applied unchanged or was it modified (if so, how)?
E.g. to fit the specific circumstances of the patient or local clinical setting</td></tr>
<tr><td>-QD3. Can you tell me the story around the use of this EE+ page for this patient?
Probe 1: What happened after you found the information?
Probe 2: What happened since then?
Probe 3: Did you have a follow-up with this patient?
(…)
If other, please explain (e.g., “no use” or “NA” or other)</td></tr>
</table>

PART E. OUTCOMES = Questions at the HIT LEVEL

-QE1. In summary, did this *EE+* page have any patient outcomes? If yes, what specific patient outcomes?

- *What was the clinical situation before you find this information?*
- *What was the clinical situation after you applied this information?*
- *Imagine that you did not find this information. Would the health of the patient have been different?*

-QE2. Just to make sure I understand correctly, can you answer to the following questions by Yes or No? This *EE+* page:

- Increased patient knowledge about health or healthcare?
- Avoided unnecessary or inappropriate treatment, diagnostic procedure or preventive intervention?
- Increased patient acceptability of treatment, diagnostic procedure or preventive intervention?
- Prevented disease or health deterioration (including acute episode of chronic disease)
- Improved patient health or functioning or resilience (the way patient faces difficulties)?

[*If needed*]

-QC1. For the **second** *EE+* page entitled (*read title*), you reported the following impacts (*read log-report*). In what specific ways did this page have the impact “(*read impact*)”?

Etc. (each impact + Application…)

PART C. REPEAT ALL QUESTIONS FOR ANOTHER SEARCH…

Finally, thank you very much and I would like to know whether you have any comment about the study, the data collection process or this interview.
