# Supplementary material for: In pursuit of a valid information assessment method for continuing education: a mixed methods study
Source: BMC Med Educ. 2013 Oct 7;13:137. doi: 10.1186/1472-6920-13-137 (PMC3842783; doi:10.1186/1472-6920-13-137)
Supplement: Additional file 2: — Definitions of IAM items. [file 1472-6920-13-137-S2.pdf]

Additional file 2

Definitions of IAM items

ITEM	ITEM DEFINITION
**Search Objective**	
1. To address a clinical question/problem/decision about a specific patient	A search to solve a problem in clinical care such as information on etiology, diagnosis, investigations, interpreting test results, drug information, disease staging and prognosis.
2. To fulfill a personal educational objective	A search for the purposes of educating oneself.
3. To satisfy curiosity or personal interest	A search for gathering general information for the purposes of personal interest and general knowledge.
4. To look up something I had forgotten	A search for previously known information which was forgotten
5. To share information with a patient or their family or home health aides	A search to share information with patients, their families or caregivers at home.
6. To exchange information with other health professionals	A search to share information with other health professionals.
7. To manage aspects of patient care with other health professionals	A search to plan/manage/coordinate tasks related to patient care with other health professionals.
**Cognitive Impacts**	
1. My practice was (will be) changed and improved	A change in decision-making with respect to a patient (or a commitment to change).
2. I learned something new	A change in knowledge.
3. This information confirmed I did (I am doing) the right thing	A reinforcement of decision-making.
4. I was reassured	A state of increased comfort.
5. I was reminded of something that I already knew	A prompt that stimulated memory.
6. I was dissatisfied	Dissatisfaction because an information need is not satisfied.
7. There is a problem with the presentation of this information	Dissatisfaction because of issues with the content such as too much information, too little information or format issues with the information;
8. I disagree with the content of this information	Disagreement with the content of the information.
9. This information is potentially harmful	A situation where information is perceived to be harmful.

	Use of Information for a Specific Patient	Definition
1.	As a result of this information I did (or will) manage this patient differently.	Information directly modifies a management plan for a specific patient.
2.	I hesitated between options for this patient, and I used this information to justify a choice	Information used to make a choice between two or more options for a specific patient.
3.	I did not know what to do, and I used this information to justify a choice	Information used to make a decision in the absence of an initial plan, for a specific patient.
4.	I used this information to better understand a particular issue related to this patient	Information used to change "awareness, thinking, or understanding of specific issues".
5.	I thought I knew what to do, and I used this information to be more certain about the management of this patient	Information sustaining the planned management in the absence of an initial plan, for a specific patient.
6.	I used this information to persuade a specific patient or other health professionals to make changes	Information used to persuade others for modifying action.
7.	I used (will use) this in a discussion with this specific patient or other health professionals	Information used to promote discussion with a specific patient or health professionals about a specific patient.
	**Patient health outcomes**	**Definition**
1.	This information helped to increase this patient's knowledge(or their family or home health aides) about heath or healthcare	Increased knowledge of health and health care enables individuals to maintain or improve their own health, as well as the health and well-being of others;
2.	This information helped to avoid (will help to avoid) unnecessary or inappropriate treatment, diagnostic procedure, preventative interventions or referral to another specialist, for this patient	Appropriateness of place and provider reflects primary health care's key roles: providing the right service by the right person at the right time, and acting as a source of first-contact care and referral to specialty services
3.	This information helped to decrease (will help to decrease) patient's worries about a treatment, diagnostic procedure or preventative intervention	Patient satisfaction with health care provided, including decreasing patient anxiety;
4.	This information prevented (will help to prevent) a disease or worsening of disease for this patient	Reduced risk, duration and effects of acute and episodic conditions and reduced risks and effects of continuing or chronic conditions;
5.	This information helped to improve (will help to improve) this patient's health status or functioning or resilience (i;e;, ability to adapt to significant life stressors)	Improved patient health, functioning and resilience (i.e., ability to adapt in the face of trauma or ongoing significant life stressors)
